# Larval rockfish growth and survival in response to anomalous ocean conditions

**DOI:** 10.1038/s41598-023-30726-5

**Published:** 2023-03-11

**Authors:** H. William Fennie, Kirsten Grorud-Colvert, Su Sponaugle

**Affiliations:** 1 Department of Integrative Biology, Oregon State University, Corvallis, OR 97331 USA; 2 Hatfield Marine Science Center, Oregon State University, Newport, OR 97365 USA; 3 Present Address: NOAA Fisheries Service, Fisheries Resource Division Southwest Fisheries Science Center, La Jolla, CA 92037 USA; 4 Present Address: Institute of Marine Sciences, University of California Santa Cruz, Santa Cruz, CA 95605 USA

**Keywords:** Population dynamics, Climate-change ecology

## Abstract

Understanding how future ocean conditions will affect populations of marine species is integral to predicting how climate change will impact both ecosystem function and fisheries management. Fish population dynamics are driven by variable survival of the early life stages, which are highly sensitive to environmental conditions. As global warming generates extreme ocean conditions (i.e., marine heatwaves) we can gain insight into how larval fish growth and mortality will change in warmer conditions. The California Current Large Marine Ecosystem experienced anomalous ocean warming from 2014 to 2016, creating novel conditions. We examined the otolith microstructure of juveniles of the economically and ecologically important black rockfish (*Sebastes melanops*) collected from 2013 to 2019 to quantify the implications of changing ocean conditions on early growth and survival. Our results demonstrated that fish growth and development were positively related to temperature, but survival to settlement was not directly related to ocean conditions. Instead, settlement had a dome-shaped relationship with growth, suggesting an optimal growth window. Our results demonstrated that the dramatic change in water temperature caused by such extreme warm water anomalies increased black rockfish growth in the larval stage; however, without sufficient prey or with high predator abundance these extreme conditions contributed to reduced survival.

## Introduction

As climate change progresses, the oceans are absorbing ~ 90% of the heat attributed to global warming^1^. There is evidence that much of the ocean has been warming for decades (+ 0.65 °C since 1960)^2^, and increasing ocean temperatures have the potential to dramatically impact marine organisms, populations, and ecosystems^3^. Understanding the impacts of changing ocean conditions on marine populations and ecosystems is essential to the development of “climate-ready” management strategies^4^ to support the ecosystem structure and productive fisheries these species underpin. As fish population dynamics are driven by variable survival through fish early life stages^5^, and larval mortality increases with elevated temperatures^6,7^, climate change is likely to alter survival during early life^8^ and thus overall population abundances.

The larval phase is a critical period in the development of many marine fish species; however, relatively little is known about the processes leading to successful larval growth and recruitment to adult populations. Oceanographic conditions dictate water temperature and influence larval dispersal and food availability, all of which affect the early growth and survival of larval fishes^6,9–11^. Variation in early growth is an important factor explaining variability in year-class strength of fished species as small changes in growth and survivorship can lead to large fluctuations in the magnitude of recruitment to the population and thus to the fishery^12^. In addition, larval experience can influence survival and performance in later life stages through carryover effects, such as rapid larval growth contributing to increased juvenile survival following settlement to reefs^13,14^. Thus, to predict population dynamics of fish populations in future oceans, we need to understand how early life stages will grow and survive in those new environmental conditions. One “natural experiment” that enables the collection of valuable field data is the recent occurrence of large-scale marine heatwaves that mimic future ocean conditions.

Eastern boundary current systems support highly productive fisheries and are affected by ocean and land warming patterns^15–17^. The California Current Large Marine Ecosystem (CCLME) along the eastern boundary of the North Pacific Ocean is characterized by high but variable seasonal productivity. In winter, low-pressure systems in the north Pacific generate storms that create turbulent mixing in the upper water column and poleward winds drive onshore Ekman transport moving upper layers of water towards shore, creating downwelling conditions. During the spring and summer, warming of the continental landmass creates a low pressure in contrast to the high pressure formed by the cooler air over the ocean. This pressure gradient generates equatorward winds that drive offshore Ekman transport, leading to the upwelling of cold nutrient-rich water along the coast^18^. Many fish species time their reproduction to minimize offshore transport^19^ and to take advantage of the high productivity during the upwelling season^20,21^. Warming threatens to alter reproductive phenology and early survival, and there is mounting evidence that climate change is already impacting the CCLME by altering the distribution and phenology of fishes^22,23^, as well as the intensity of upwelling winds^24^. In addition, the CCLME recently experienced extreme conditions during a large marine heatwave (2014–2016) that affected the ecosystem and its inhabitants^25,26^. Understanding how anomalous oceanographic conditions in the CCLME impact the early life stages of fishes and their survival through recruitment is important for managing fisheries in the face of changing ocean conditions.

Rockfishes (genus *Sebastes*) are a group of economically and ecologically important fish species found throughout the CCLME. Adult rockfish have comprised the backbone of the US west coast commercial bottom fishery since the 1940s^27^ while the pelagic juvenile stages are important prey for a variety of predators in the CCLME^28–31^. Rockfishes are characterized by their long-life spans, high fecundity, ovoviviparity, and highly variable survival during their early life stages. Females give birth to pelagic larvae that develop in the plankton for up to several months before metamorphosing into juveniles that eventually settle to benthic adult habitats^21,32^. Environmental factors during the rockfish pelagic phase are thought to regulate year class strength^33^. Recruitment variability of different rockfish species in California has been linked to different conditions: a combination of zooplankton prey abundance and reduced offshore transport^34^, the Northern Oscillation Index^35^, northern copepod biomass (potentially important prey and an indicator of increased equatorward transport of productive northern waters)^36^, regional productivity^37^, and, prior to the marine heatwave, sea level height anomalies^38^. During the 2014–2016 marine heatwave, the relationship between sea level height anomaly and rockfish recruitment collapsed and a new “spiciness index” was computed. Spiciness is a measure of the relative contribution of Pacific Subarctic and Pacific Equatorial water to the upper 100-400 m of the CCLME and the spiciness index effectively explained variability in rockfish recruitment in California, including during the anomalously warm conditions^39^. Spicy conditions indicate relatively low Pacific Subarctic water and high Pacific Equatorial water, thus a warm, salty, and low oxygen signal. In contrast, minty conditions signal the presence of cold, fresh, and highly oxygenated water. While these anomalous conditions enabled the refinement of our understanding of how changing ocean conditions affect the recruitment of rockfish in California, mechanisms underlying these patterns remain largely unknown. Rockfishes also recruit to more northern areas along the Oregon and Washington coasts and up to British Columbia^40,41^, in some cases comprising a substantial commercial and recreational fishery. Oceanographic conditions differ in these northern portions of the CCLME^42^ and little is known regarding how oceanographic conditions influence the growth and survival of these species.

We used a time-series of settlement-stage juvenile black rockfish (*Sebastes melanops*) from 2013 to 2019 along the central coast of Oregon to investigate the influence of oceanographic variability on fish early life history traits and survival (Table 1). This time-series included the extreme conditions of 2014–2016, allowing for a glimpse into how future ocean conditions may impact black rockfish early life. Otolith microstructure analysis was used to quantify black rockfish early life history traits while calculation of settlement rate (i.e., catch per unit effort) to nearshore monitoring devices provided an estimate of survival through the pelagic period to the point of nearshore settlement. We examined how early growth and settlement magnitude were related to a variety of basin-scale, regional, and local oceanographic indicators, to shed light on what we can expect in future oceans.

**Table 1:** Otolith sample sizes used to estimate early life history traits of black rockfish (*Sebastes melanops*).

Year	Sample size (n)	Recruitment window
2013	15	June 6–July 17
2014	34	May 28–June 25
2015	1 + (5)	May 18–June 29
2016	32	May 17–June 29
2017	27	May 18–June 27
2018	29	May 16–July 17
2019	32	May 10–July 5

All samples from central Oregon except where parentheses indicate individuals from the southern Oregon SMURFs (233 km away, also in the Northern California Current). The recruitment window indicates the dates used to calculate settlement rate.

## Results

### Oceanography during the larval phase of rockfish

**Figure 1:**
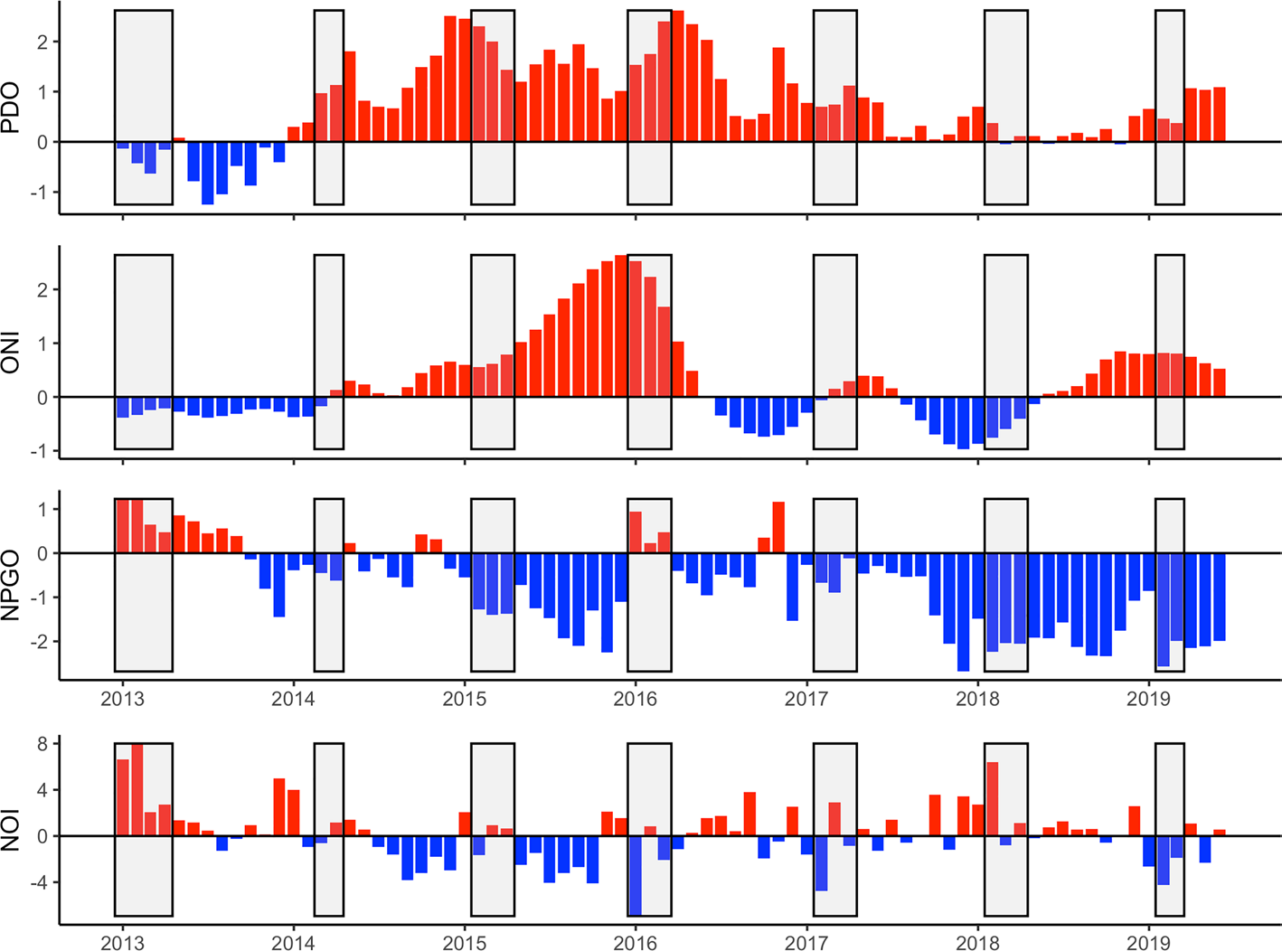
Monthly mean basin-scale climate indices. From top to bottom: Pacific Decadal Oscillation (PDO), Ocean Niño Index (ONI), North Pacific Gyre Oscillation (NPGO), and the Northern Oscillation Index (NOI). Gray rectangles indicate the larval period for each annual cohort of black rockfish (*Sebastes melanops*)–birthdate distribution plus one month.

Larval black rockfish experienced a wide diversity of conditions from 2013 to 2019. On a basin scale, the Pacific Decadal Oscillation (PDO), a measure of sea surface temperature (SST) throughout the northeast Pacific, during the larval period was strongly negative during 2013 and somewhat neutral during 2018, while Ocean Niño Index (ONI), a measure of equatorial upper water temperature and indicator of El Niño events, was negative in 2013 and 2018, indicating enhanced coastal upwelling and cooler water temperatures during larval development (Fig. 1). In contrast, both PDO and ONI were positive during the larval rockfish windows in 2014, 2017, and 2019, and strongly positive in 2015–2016, indicating that fish experienced reduced upwelling and warmer water temperatures. The marine heatwave began to impact the CCLME in 2014, which is apparent in the transition from negative PDO and ONI in 2013 to positive in 2014, but the dramatic effects of the heatwave did not impact the northern CCLME until after the larval period. However, both the PDO and ONI shifted to positive in winter of 2014 ahead of the heatwave, indicating that larvae in 2014 developed in warm and reduced upwelling conditions. Likewise, the North Pacific Gyre Oscillation (NPGO), which is a measure of upwelling and horizontal transport, was positive in 2013 and 2016, negative in 2014 and 2017, and strongly negative in 2015, 2018, and 2019 (Fig. 1). The Northern Oscillation Index (NOI), which is associated with trade wind and upwelling strength, was positive during the larval stage in 2013 and 2018, near the long term mean for 2014 and 2015, and negative in 2016, 2017, and 2019 (Fig. 1).

Regional oceanographic indices varied across years. Meridional winds were poleward in 2013–2014 as well as 2016–2018, strongly equatorward in 2015, and moderately equatorward in 2019. The biologically effective coastal upwelling transport index (BEUTI) was negative during the larval stage from 2013 to 2018 and turned positive in 2019 (Table 2).

**Table 2:** Oceanographic indicators averaged over the larval period (birthdate distribution plus 1 month) of black rockfish (*Sebastes melanops*) used in the partial least squares regressions with mean larval growth and settlement rate.

Year	PDO	ONI	NPGO	NOI	BEUTI	Mwind	S.copepod	SST	Spicy
2013	− 0.338	− 0.375	0.888	4.85875	− 0.259	0.030	− 0.035	9.642	− 0.654
2014	1.05	− 0.115	− 0.536	0.278	− 1.32	1.430	− 0.187	10.595	− 0.384
2015	1.913	0.567	− 1.349	− 0.036	− 0.023	− 1.2	0.401	11.406	− 0.395
2016	1.893	2.067	0.547	− 2.725	− 2.481	4.836	0.348	11.461	− 0.266
2017	0.853	0.03	− 0.559	− 0.7916	− 2.805	3.475	0.405	10.537	− 0.086
2018	0.143	− 0.683	− 2.112	2.222	− 0.178	0.432	0.182	10.197	− 0.215
2019	0.415	0.715	− 2.282	− 3.0745	0.22	− 0.047	0.155	10.441	− 0.161

Pacific Decadal Oscillation (PDO), Ocean Niño Index (ONI), North Pacific Gyre Oscillation (NPGO), Northern Oscillation Index (NOI), regional: biologically effective upwelling transport index (BEUTI), meridional wind velocity (Mwind), and local oceanographic conditions: southern copepod biomass anomaly (S.copepod), sea surface temperature (SST), and the spiciness index (Spicy).

**Figure 2:**
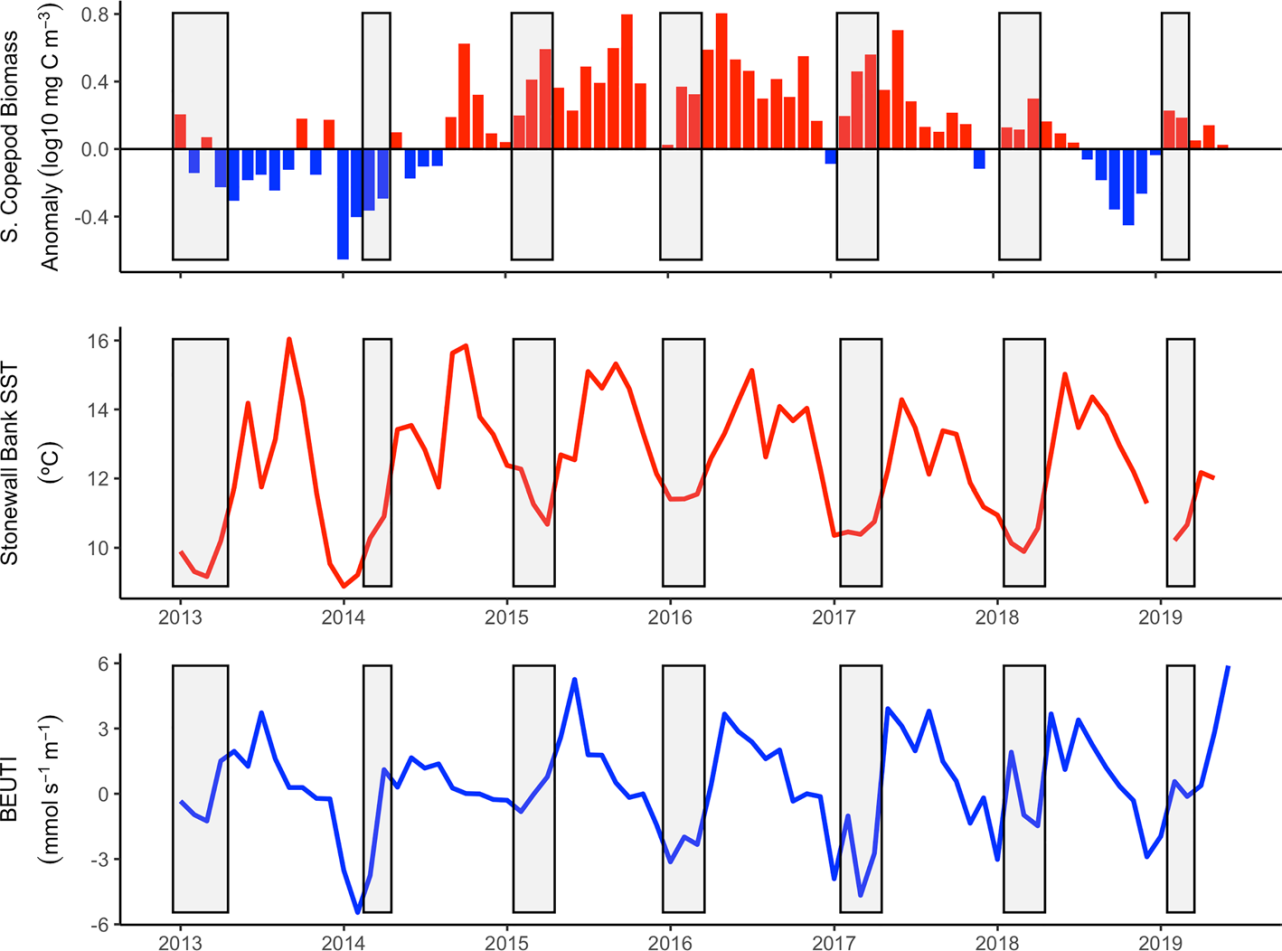
Monthly mean local climate indices. From top to bottom: Southern Copepod Biomass Anomaly Index, sea surface temperature (SST) at Stonewall Bank, OR, and biologically effective coastal upwelling transport index (BEUTI) at 45°N. Gray rectangles indicate the larval period for each annual cohort of black rockfish (*Sebastes melanops*)–birthdate distribution plus one month.

Local oceanographic indicators aligned with the basin-scale indices. The southern copepod biomass anomaly trended with the PDO and ONI and was negative during the larval period in 2013–2014, strongly positive in 2015–2017 as the warm water mass moved into the northeast Pacific, and weakly positive in 2018–2019 (Fig. 2). SST generally tracked the PDO/ONI values with cold temperatures in 2013, markedly warmer temperatures in 2014–2017, cooling off in 2018, before warming slightly in 2019 (Fig. 2). The spiciness index suggested that source waters of the CCLME throughout this time series were dominated by Pacific Subarctic water, with the greatest influence in 2013–2016 and a relatively smaller contribution in 2017–2019 (Table 2).

### Rockfish early life history traits & settlement rate

**Figure 3:**
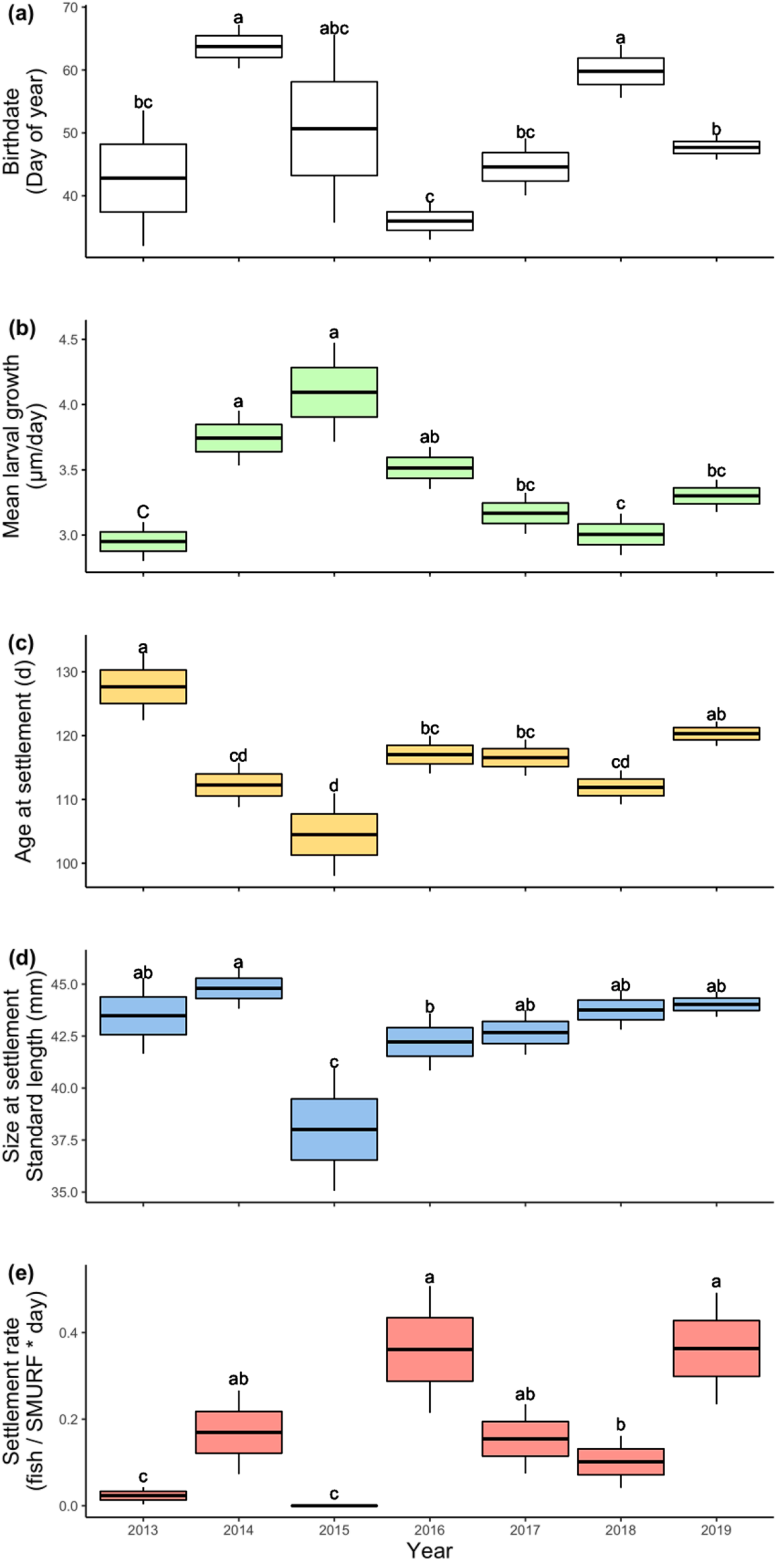
Mean (thick line), standard error (box) and 95% confidence interval (whisker) of (**a**) birthdate distribution, (**b**) age at settlement, (**c**) mean larval growth rate, (**d**) size at settlement, and (**e**) settlement rate of black rockfish (*Sebastes melanops*). Letters above boxplots represent Tukey-HSD post-hoc comparisons. Shared letters are not significantly different.

There was significant interannual variability in black rockfish early life history traits. Birthdates varied by year (F_6, 168_ = 22.57, *p* < 0.001) with fish born earliest in 2016 with a mean birthdate of February 5 and latest in 2014 and 2018 with a mean date of March 6 (28 days later; Fig. 3a). Mean larval growth differed significantly across years (F_6,168_ = 13.32, *p* < 0.001) with growth ~ 27% faster in the anomalously warm years of 2014–2016 than growth in cool years of 2013 and 2018 (Fig. 3b). Age-at-settlement varied significantly across years (F_6, 168_ = 11.25, *p* < 0.001) and mirrored growth rate as settlers were typically younger in warm years when growth was fastest (2014–2016) and oldest in 2013 (8% older) when growth was slowest and water temperatures were coldest (Fig. 3c). Size (SL) also varied significantly among years (F_6,168_ = 6.102, *p* < 0.001): Fish were largest in 2014 and smallest in 2015 (15% smaller; Fig. 3d). There was significant interannual variability in settlement rate (Table 3) with highest settlement in 2016 and 2019 and lowest settlement in 2013 and 2015 (Fig. 3e).

**Table 3:** Results of generalized linear model with negative binomial distribution of interannual black rockfish settlement.

Variable	df	Deviance	Residual df	Residual Deviance	Pr(> Chi)
Null			227	346.41	
Year	6	144.37	221	202.04	< 0.0001

### Relationship between larval growth and oceanography

**Figure 4:**
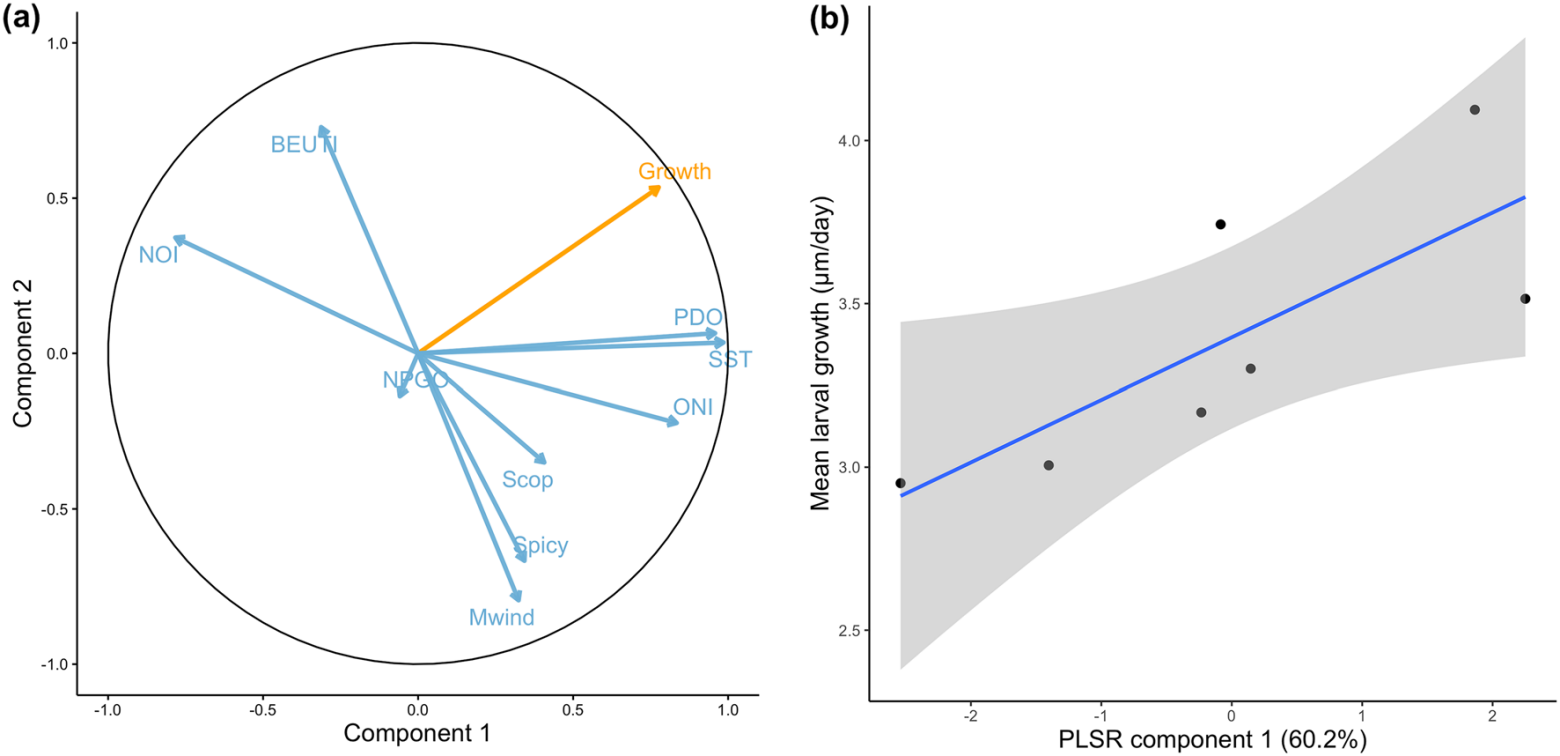
Partial least squares regression (PLSR) correlation (a) radar plot of oceanographic indices (blue) and mean larval growth of black rockfish (*Sebastes melanops*) (orange) based on 7 years of settlement-stage fish. (**b**) Linear regression of mean larval growth as a function of PLSR component 1; gray area is 95% confidence interval. Indices as listed in Figs. 1 and 2.

Rockfish larval growth rate is largely driven by ocean temperature. The first two components of the partial least squares regression analysis explained 88.8% of variability in mean larval black rockfish growth, with the first component explaining 60.2% of the variability (Fig. 4a). There was a strong positive relationship between mean larval growth and the first PLSR component, which was driven mainly by PDO and SST (Table 4), indicating that growth was faster in years with warmer water (*p* = 0.04; R^2^ = 0.60; Fig. 4b). The second component captured 28.6% of the variability but mean larval growth was not significantly correlated with this component (*p* = 0.22).

**Table 4:** Summary of the partial least squares regression analysis between basin scale: Pacific Decadal Oscillation (PDO), Ocean Niño Index (ONI), North Pacific Gyre Oscillation (NPGO), Northern Oscillation Index (NOI), regional: meridional wind velocity (Mwind), biologically effective upwelling transport index (BEUTI), the spiciness index (Spicy), and local oceanographic conditions: sea surface temperature (SST), and southern copepod biomass anomaly (S.copepod) and mean larval growth of black rockfish (*Sebastes melanops*).

Predictor	Weight^2^	Correlation	Loading
**PDO**	**0.412**	**0.958**	**+**
**SST**	**0.369**	**0.985**	**+**
ONI	0.103	0.834	**+**
NOI	0.097	−0.783	−
Mwind	0.011	0.324	**+**
S.copepod	0.006	0.407	**+**
NPGO	0.005	−0.059	−
Spicy	0.002	0.342	**+**
BEUTI	0.0001	−0.313	−

Weight^2^ indicates the contribution of each variable to the variability explained by Axis 1. Correlation is between mean larval growth and each indicator variable. Loading indicates the direction of the relationship between the explanatory variable and mean larval growth. Significant predictor variables that explained more than 20% of variability in Axis 1 are in bold.

There was a weak relationship between black rockfish settlement and the same suite of oceanographic and early life history variables. The first two components of the partial least squares regression explained 89.9% of the variability in black rockfish settlement. The first component explained 51.3% of the variability in settlement while the second component explained 38.6% of the residual variation in settlement; however, settlement was not significantly correlated with either component (*p* = 0.07 and *p* = 0.14, respectively).

### Relationship between growth, timing of metamorphosis, and settlement

**Figure 5:**
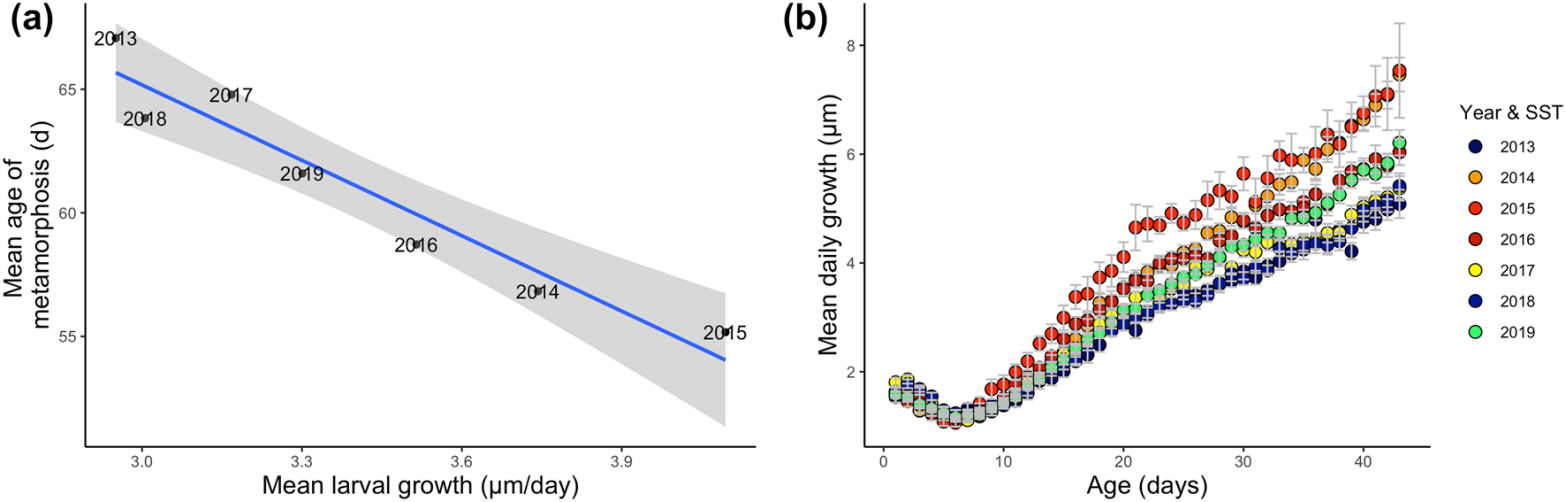
(**a**) Linear regression of the timing of metamorphosis (transition between larval and pelagic juvenile stages) of larval black rockfish (*Sebastes melanops*) and larval growth rate. Gray area is 95% confidence interval. (**b**) Mean daily larval growth (otolith increment widths) for each of seven annual cohorts of black rockfish. Colors correspond to relative mean water temperatures encountered by larvae with higher temperatures in reds and lower temperatures in blues.

**Figure 6:**
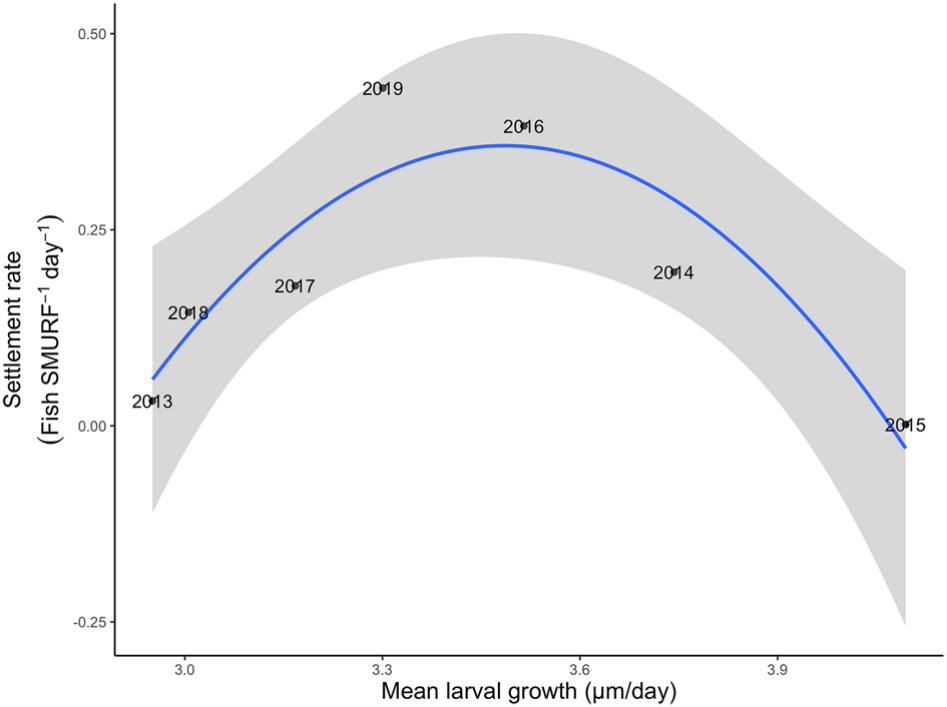
Multiple regression of mean settlement rate of black rockfish (*Sebastes melanops*) to coastal Oregon as measured in replicate Standardized Monitoring Units for the Recruitment of Fishes (SMURFs) over 7 years as a function of mean larval growth and mean larval growth^2^. Gray area is 95% confidence interval.

The timing of larval black rockfish metamorphosis to pelagic juveniles was negatively related to mean larval growth (R^2^ = 0.92, *p* < 0.001), suggesting that rapid growth enables a faster transition from larvae to juvenile (Fig. 5). In contrast, annual settlement magnitude of black rockfish was not a linear function of mean larval growth, but instead was dome-shaped, with settlement highest at an intermediate value of mean larval growth (Adj. R^2^: 0.72, F_2,4_ = 8.88, *p* = 0.03; Fig. 6).

## Discussion

### Marine heatwaves and rockfish in the California Current

Over the 7-yr period of 2013–2019, the CCLME experienced extremely variable oceanographic conditions. The advent of the large anomalous marine heatwave from 2014 to 2016 resulted in the warmest three-year period in the recorded history of the CCLME^43^. These years consisted of strongly positive PDO values, a very strong El Niño (2015–2016), and strongly negative NPGO values, triggering ecosystem-wide changes in the taxa distributions, reproductive phenology^22,23^, larval fish abundance, and community structure in the CCLME^44^. These extreme conditions also changed our previous understanding of the relationship between the physical environment and rockfish recruitment in California^39^.

In Oregon, the extreme event of a marine heatwave resulted in changes throughout the planktonic food web. In the phytoplankton community, diatom abundance and diversity decreased and dinoflagellate diversity spiked^45^. The zooplankton community experienced a reduction of copepod and euphausiid biomass, but increased in copepod species richness as a diversity of southern and offshore species moved into the system^46^. Additionally, micronekton and macrozooplankton communities underwent a shift from crustacean to gelatinous zooplankton dominance^47^. In 2015, the plankton community experienced a dramatic increase in the hydromedusa *Aequorea victoria*, but by 2016, the pelagic tunicate *Pyrosoma atlanticum* dominated the system^47,48^. These changes to the base of the food web led to changes in the diets of many forage fish off the Oregon coast with gelatinous zooplankton becoming a major proportion of the prey consumed^49^.

Rockfish are an important component of this ecosystem, but few studies have examined how they have and will respond to warming-induced ecosystem changes in the northern CCLME. Pelagic juvenile rockfish are considered to be a forage species as they link large predators to lower trophic levels^28^. The diets of pelagic juvenile rockfish are typically dominated by copepods, euphausiids, and amphipods^50,51^; however, not much is known about larval rockfish diets and how they may change with oceanographic conditions^52^. Abundance of larval rockfish has been positively correlated with the multivariate ENSO index and eastward Eckman transport^53^. Additionally, settlement of several rockfish groups has been shown to increase with downwelling conditions prior to settlement while the settlement of others increased with higher SST, though the group containing black rockfish did not have a strong response to either of these conditions^40^.

Marine heatwaves cause major changes to oceanic ecosystems and are expected to become more frequent as oceans continue to warm^54^. These events provide the opportunity to examine relationships between extreme conditions and settlement patterns as a precursor to refining our predictions of climate change effects on future fish populations. By examining a 7-yr time series of settlement that included multiple years of anomalous conditions, we were able to tease apart the effect of environmental conditions on the growth, development rate, and survival of juvenile black rockfish, together informing our understanding of the mechanisms underlying patterns of recruitment.

### Variation in rockfish traits

For black rockfish settling to coastal Oregon, this period of highly variable oceanographic conditions led to interannual variability in juvenile black rockfish early life history traits. Birthdate distributions varied across years but were not associated with ocean conditions. However, there were clear linkages across other traits: growth and development were strongly affected by temperature, leading to faster growth, rapid development, and earlier settlement in warm years relative to cool years. Because size at the time of settlement is a function of both growth rate and time spent growing at that rate, size-at-settlement was largely conserved across years (~ 42 mm SL) except in 2015 when growth was highest, and size-at-settlement was significantly smaller. Variability in growth is commonly attributed to variable temperatures, and work on temperate species shows that warmer temperatures increase early growth^55^ and weight-at-age of species such as Atlantic cod (*Gadus morhua*)^56^. Studies of tropical fishes have shown that water temperature encountered by larvae during early life not only influences their growth, but also their larval duration and size-at-settlement^57,58^.

Temperature plays a major role in black rockfish larval growth. Early growth was strongly positively associated with PDO and SST, indicating faster growth in warmer water. Laboratory studies of larval and juvenile rockfish growth demonstrate that, as long as fish have sufficient access to food, growth rate increases with increasing temperature^59,60^. Field studies confirm that larval growth increases with increasing temperature for rockfish off the coast of central California^61^ and off of Washington, USA^62^. The fish used in our study were those that had successfully navigated the pelagic larval period and were collected at the time they transitioned to the nearshore. Because these fish had survived this high mortality stage, by definition, they were those that had enough food to survive. However, for larval fishes in general, the interplay between temperature, food availability, and growth will likely be important in determining how climate change will impact the early survival of fishes^8^ and thus adult population dynamics.

### Oceanography and rockfish settlement to central Oregon

Perhaps surprisingly, the magnitude of black rockfish settlement to central Oregon was not significantly correlated with oceanographic conditions. The two highest settlement years (2016 and 2019) were characterized by positive PDO and ONI conditions, although 2016 had much higher PDO and ONI values. These years differed substantially from each other in that relative to 2019, 2016 had an elevated southern copepod biomass anomaly, weakly positive NPGO (in contrast to strongly negative values in 2019), northward meridional winds and moderate downwelling (in contrast to southward meridional winds and upwelling in 2019), and high SST (in contrast to moderate SST in 2019). In 2015, when black rockfish settlement was lowest, PDO and ONI were positive while NPGO was negative, meridional winds were southward and there was an early pulse of upwelling, southern copepod abundance was enhanced, and SST was high. The overlapping and contrasting conditions within years of high recruitment and between years of high and low recruitment demonstrate the challenge in using oceanographic conditions in a dynamic ecosystem experiencing anomalous conditions to forecast rockfish survival.

Previous studies in California have shown that the pelagic juvenile abundance of a variety of rockfish species is related to oceanographic variability (Table SI and references therein). Juvenile black rockfish abundance has been shown to be negatively associated with sea level anomaly and nearshore temperature during the larval stage^63^. However, only one study encompassed the marine heatwave and demonstrated that the spiciness index best explained rockfish abundance throughout the time series^39^. Inclusion of the anomalous marine heatwave conditions altered our previous understanding of how pelagic juvenile abundance of a variety of rockfish species varies with ocean conditions^38,63^. For black rockfish settling to central Oregon, settlement magnitude was not significantly correlated with the spiciness index or any other oceanographic index. Instead, we found that low settlement occurred in one year of elevated water temperatures as well as in the coldest year, while high settlement occurred in two warm years. It is possible that there are regional differences in how black rockfish respond to ocean conditions as upwelling in Oregon is more intermittent than the near constant upwelling of northern and central California^42^.

The combination of the differences in Oregon oceanography and the inclusion of the marine heatwave in this settlement timeseries may explain why conditions that predict early survival of pelagic California rockfish do not reliably predict black rockfish settlement to the nearshore in Oregon. Importantly, the majority of previous studies in California have focused on the abundance of pelagic juveniles offshore and not on nearshore settlement or recruitment^37,63^. Nor do these studies provide a direct link between oceanographic variability and rockfish early life history traits. Without a mechanistic understanding of how oceanographic variability translates into variability in rockfish early life history traits we cannot anticipate how changing conditions will affect rockfish survival. Our study is one of the first to link these larval oceanographic variables with nearshore recruitment for black rockfish. Here we demonstrate that ocean conditions impact rockfish larval growth and that oceanographic variability is translated to settlement variability through larval and pelagic juvenile growth.

### Early life history traits and magnitude of rockfish settlement

The annual magnitude of black rockfish settlement to central Oregon varied predictably with larval growth, but in an unexpected way. While a combination of environmental and oceanographic variables failed to predict black rockfish settlement rate, a better predictor emerged: growth integrates the influence of the biological and physical environment on larval development. Numerous studies stress the importance of fast growth^64–66^, large size^67,68^ and rapid development^12,69^ to the survival of young fishes. We found that water temperature strongly influences larval growth and development, but interestingly, this rapid larval growth did not translate to high survival. Survival to settlement was highest at moderate growth rates in conditions that likely increased food availability and decreased predation. Our findings demonstrate that warming of the CCLME will increase black rockfish larval growth rates, but depending on how the entire ecosystem responds to warming, may lead to reduced survival to settlement.

Although growth of young black rockfish off Oregon was positively related to water temperature, fast growth in warm temperatures did not lead to high rates of settlement. In fact, settlement rate was lowest in 2015 when growth was fastest and peaked at intermediate values of larval growth. Settlement was so low in 2015 that there were barely enough fish samples to analyze. There are several possible explanations for why settlement rate was lowest in 2015 when growth was highest, and the following two explanations are most likely. First, in 2015, strong meridional winds during the larval period led to a strong early upwelling pulse and offshore transport, potentially displacing larvae offshore or beyond their retention zone^11^ which may have dramatically decreased the survival of this cohort as only a few extremely fast-growing individuals were collected at settlement. Swimming endurance in rockfish increases with increasing size and development^70^. It is possible that only the individuals that grew and developed rapidly were able to resist or overcome offshore displacement and settle to the nearshore. Second, there may have been strong growth-selective mortality^66^ where predators consumed the slowest growing individuals and only the fastest growers escaped predation and survived to the settlement stage. There is evidence of size-selective predation of larval fish by hydromedusae^71^ and faster growing fish are likely to reach larger size more rapidly, reducing the amount of time they would be vulnerable to gelatinous predators. Abundance of *A. victoria,* a hydromedusa that consumes larval rockfishes^72^, dramatically increased off the coast of Oregon in 2015^47^ and may have caused catastrophic losses to the larval black rockfish population in 2015.

While measured rates of settlement in our study were extremely low in 2015 and young rockfish may have experienced high mortality due to physical transport and/or predation by *A*. *victoria*, data from 2014 provide an interesting comparison. In 2014 when larval rockfish growth was relatively fast and settlement moderate, upwelling strength and *A*. *victoria* abundance were low, thus inconsistent with either physical transport or predator-mediated high mortality driving reduced settlement. We hypothesize that the reduction in settlement at high larval growth rates is due to a combination of high temperature and reduced food availability with high predation underlying the most extreme low survival at the highest growth rate.

Our observations of high black rockfish growth coupled with reduced survival (settlement magnitude) in anomalously warm water during 2014–2015 may be due to changes in food availability. As with most poikilotherms, rockfish growth and consumption increases at higher temperatures and, as long as there is access to sufficient prey, fish condition also increases^59,60^. However, when prey are unavailable, increased temperature leads to increased growth, but reduced condition^59^. Experiments mimicking heatwave conditions on the larvae of a tropical fish species revealed similar patterns of enhanced growth at higher temperatures with the requirement of high consumption to maintain elevated growth^73^. While larval black rockfish were growing rapidly in 2014–2015, the copepod community shifted to one dominated by offshore and southern species that are lower in quality than the northern species associated with cooler years^74^. To support higher growth in these warm, nutrient-poor waters, we hypothesize that larval black rockfish would have to increase their foraging rate, raising the likelihood of encounters with predators and reducing survival, especially in years with high predator abundance^75^. This hypothesis remains to be tested as details of how such a shift in zooplankton community translates into the diets of young rockfish is largely unknown. We found only one study of larval rockfish diets that indicated that copepod nauplii were important to larvae of a related rockfish species (*S. paucispinis*)^52^. The majority of early rockfish diet studies focus on the pelagic juvenile phase and indicate that adult copepods are important prey items^50,51^. These findings suggest that southern copepod biomass anomalies may be more relevant to juvenile rockfish survival and that examination of fluctuations in the abundance and species composition of copepod nauplii is necessary to understand how changing ocean conditions affect the diets of larval rockfish.

Changes to food-webs in future oceans remain a large challenge in our efforts to predict the resilience of fish populations. While marine heatwaves provide some indication of how both lower and upper trophic levels may shift with climate-induced changes, the consequences of shorter-term community changes may be different than long-term community changes. Thus, a deeper understanding of food webs in important ecosystems such as the CCLME will be necessary to fully understand how climate change will impact fish populations.

### Climate change and the future of fishes in the CCLME

The CCLME is already feeling the effects of climate change, making it imperative to understand future impacts to larval fish survival and subsequent population dynamics during the adult, fished life stages. Evidence of changes in the timing, intensity, and duration of upwelling in the CCLME is mounting^15–17^: upwelling is intensifying during spring but there are signals of lower upwelling overall^24,76^. Intensified upwelling could lead to enhanced productivity, or mixing could become too strong, transporting nutrients, phytoplankton, and larval fishes out of nearshore surface waters^10^. In addition, the southern portion of the CCLME is experiencing earlier seasonal warming, and many fish species, including several rockfishes, are experiencing shifts in their reproductive phenology to earlier in the year^22^. Coinciding with the large marine heatwave in the northern CCLME, reproduction of many fish species is occurring earlier and farther north than previously observed^23^. Shifts in both the timing of reproduction and the onset of seasonal productivity may become detrimental to early fish survival in the CCLME. Finally, the warm period of 2014–2016 revealed the sensitivity of the northern CCLME plankton community to changing ocean conditions^45–47,49^.

Our results demonstrated that the dramatic change in water temperature caused by such extreme warm water anomalies may increase black rockfish growth in the larval stage, but without adequate prey availability or with increased predator abundance, settlement magnitude may plummet. In our 7 year study, anomalously warm oceanographic conditions increased black rockfish larval growth, but we hypothesize that poor quality of copepod prey during the same period required increased feeding activity, leading to higher predator encounter rates, and diminished pelagic juvenile rockfish survival. The balance between constraints operating in different stages will determine the degree to which growth-related processes contribute to successful population replenishment. By integrating environmental variability (temperature, abundances of prey and predators) the examination of fine-scale individual and cohort-specific growth enables a view into the mechanisms underlying population predictions in a changing ocean.

## Methods

### Oceanographic conditions

To characterize the ocean conditions experienced by larval black rockfish during the pelagic stage we assembled a variety of basin- scale, regional, and local oceanographic data from the California Current Integrated Ecosystem Assessment data portal (https://www.integratedecosystemassessment.noaa.gov/). To examine the influence of broadscale oceanographic patterns on larval black rockfish, we obtained monthly values of periodic climate patterns: the PDO, the ONI, the NPGO, and the NOI. We included BEUTI as a regional measure of upwelling strength and productivity at 45^o^N. We also examined the local oceanographic indicator of southern copepod biomass anomalies from the Newport Hydrographic (NH) Line (off Newport, Oregon): southern copepods originate in central and southern California or offshore waters in the California Current and are transported to the northern California Current during winter. There is also a northern copepod biomass anomaly, but these larger, cold-water associated copepods with greater lipids and thus nutritional value are typically not abundant in winter when rockfish are larvae^77^. The copepod data were collected by NOAA along the NH line every 2–4 weeks throughout the study period^46^. Additionally, we included SST and meridional wind data from buoy 46050 (located 20 nmi west of Newport, OR). Data from 2019 were incomplete so we used a regression between February-May SST data from buoy 46050 and the buoy located in Yaquina Bay (SBEO3, Newport, OR) to determine the missing offshore 2019 SST values (F_1,16_ = 96.55, *p* < 0.0001, R^2^ = 0.86). Finally, we included the spiciness index for the 26.0 potential density isopycnal as a proxy of the relative contribution of Pacific Subarctic and Equatorial Pacific water to the upper waters (100–400 m) of the CCLME (sensu Schroeder et al*.,*^39^). For all these indices, we refined the values of interest by using the birthdate distribution for each juvenile rockfish cohort (see below) to compute the average monthly mean for the oceanographic data over the period fish were in the plankton (i.e., ~ 2 months from each cohort’s mean birthdate).

### Fish collection

Juvenile black rockfish were collected in standard monitoring units for the recruitment of fishes (SMURFs; Ammann^78^). These SMURFs were located in nearshore waters 16 km north of Newport, OR between Cape Foulweather and Otter Rock (see Ottmann et al*.*^40^). Eight SMURF moorings were deployed in ~ 15 m of water and SMURFs were attached 1 m below the surface. SMURF moorings were anchored in sandy habitat > 390 m from shore and well offshore of rocky reefs and kelp canopy habitats to ensure fish collected in the SMURFs were those transitioning from their pelagic to nearshore life stage. Each mooring was a minimum of 300 m apart and sampled every ~ 14 days. Fish were collected by two snorkelers using a benthic ichthyofauna net for coral and kelp ecosystems (BINCKE, Anderson & Carr^79^) to engulf a SMURF, remove it from the mooring, and bring the sample to a boat where fishes were rinsed out of the SMURFs. Following American Veterinary Medical Association guidelines for euthanasia of animals (2013), fish were euthanized with a lethal dose (2 mM) of tricaine methanesulfonate (MS-222) buffered with sodium bicarbonate (6 mM), observed for 10 min after cessation of opercular movement and then immersed in ice to ensure euthanasia, before being transported to the lab for measurement and dissection. For the purposes of determining age-at-settlement, we assumed fish that appeared in SMURFS arrived the day they were collected. Juvenile black rockfish were collected under National Marine Fisheries Service permit #18,058 and all procedures, including the ethical treatment of juvenile fishes, were approved and conducted in accordance with Oregon State University Institutional Animal Care and Use Committee protocol #4573, and followed recommendations from the ARRIVE Guideline^80^.

### Sample processing

Juvenile black rockfish were distinguished from similar looking yellowtail rockfish using pectoral fin ray counts, pectoral fin pigmentation, and dorsal coloration patterns^81^. We used digital calipers to measure each fish to the nearest 0.1 mm standard length (SL) before otoliths were dissected and removed for microstructure analysis.

To examine interannual variability of early life history traits we dissected and processed the otoliths of up to n = 34 juveniles per year (Table 1). Recruitment was very low in 2015 such that only 1 black rockfish recruited to the SMURFs deployed near Newport, OR. We used all (n = 5) black rockfish that recruited to SMURFs in southern Oregon (233 km away; see Ottmann et al.^40^) to increase our sample size for this year. We recognize that there are likely local differences in the oceanographic conditions between the two juvenile settlement monitoring locations; however, these two locations are both part of the Northern California Current and experience the same broad-scale oceanographic conditions: downwelling and storm driven vertical mixing in the winter and intermittent upwelling during the spring/summer^42^. Additionally, the impact of the largest marine heatwave ever recorded^82^, extending from Alaska to Southern California (~ + 3 °C over 1000 s of km), on the physical and biological oceanography of the California Current System during this period dwarfed local variation. Similarly, intra-annual variability in growth for the fish in central and southern Oregon is small relative to interannual differences in growth based on dramatically different oceanographic conditions (Figs. S1 & S2). Daily growth increments have been validated for juvenile black rockfish^83^, so otolith increment counts can be used to estimate age, and widths between successive otolith increments can be used as a proxy for somatic growth^37,84^. We embedded sagittal otoliths in Crystalbond thermoplastic resin (Electron Microscopy Science) and used lapping paper to polish otoliths along the sagittal plane. Otoliths were read at 400 × using a compound microscope equipped with polarized transmitted light, and increments were interpreted using image analysis software (ImagePro v.9.0). Following standard procedures^84,85^, we obtained otolith increment counts and measurements of daily increment widths to estimate the age and daily growth of each individual. Additionally, we used the presence of secondary growth, shown as primordia in the otolith, to determine the initiation of metamorphosis from the larval to pelagic juvenile stage^86^. Because juvenile black rockfish were frequently older than 100 days, it was difficult to completely encompass the core and the edge of the otolith in the same plane. We captured two otolith images: the first included the edge of the otolith with a little material remaining above the plane of core, after which the otolith was polished to the plane of the core, producing the second image. This second image always contained the core, was readable to at least the secondary growth primordia, and was used to estimate daily larval growth rate. Occasionally, this final polishing resulted in the loss of some edge material, but the use of both images during microstructure reading minimized the loss of information. We created transects from the core to the edge of each of these images, aligning these transects by otolith landmarks. This allowed us to combine the two transects, confirming overlapping regions, and create complete core-to-edge increment counts. Each otolith was read blind two independent times by the same person (HWF) and if the ages differed by > 5%, it was read a third time. If no two reads were within 5% of one another, the otolith was excluded from further analysis. For reads within 5% of each other, one read was randomly selected for further analysis. There was a significant positive relationship between the residuals of the radius-at-age and size-at-age of surviving rockfish (*F*_1,173_ = 110.2, *p* < 0.0001, R^2^ = 0.39), confirming that otolith radius and otolith increment width could be used as proxies for size and growth, respectively^87^.

### Calculation of settlement rate

We used settlement rate of black rockfish to nearshore reefs as metric of survival through the vulnerable early life stages and to understand how early survival is modified by interannual variability in oceanographic conditions. Year class strength for most California Current fish species is set after hatching/parturition, due to high mortality during early life history stages, so larval abundances are not considered reliable sources of data on recruitment dynamics; however, the pelagic juvenile abundance of several species of rockfish in the Central California Current are correlated with recruitment to the fishery^33,38,88^. Additionally, rockfish can produce strong recruitment classes despite low spawning stock biomass, therefore juvenile rockfish abundance is our best estimate of recruitment variability^88^. Black rockfish typically settle to nearshore rocky reefs and kelp beds between May and July^40^. SMURF moorings were not always deployed before the start of this window due to weather and logistical issues with deployments. To standardize annual settlement to SMURFs we restricted our analysis to the period beginning May 10 and used the last day black rockfish were collected in SMURFs each year as the end of the settlement window for that year. Settlement rate was calculated as the average number of fish per SMURF divided by the sampling interval (days between SMURF retrievals), which was then averaged for each year.

### Data analysis

We tested for interannual differences in early life history traits (birthdate, mean larval growth, and age-at-settlement) using one-way ANOVA as the error in these traits were normally distributed. We used Tukey-HSD to examine pairwise differences in early life history traits. Birthdates were calculated by subtracting fish age from the day the fish were collected. Because there was inter- and intra-annual variability in the length of the pelagic larval period (i.e., pelagic larval duration) and some fish began to metamorphose into pelagic juveniles when they were 44 days old, we limited the larval period for analysis to 0–43 days old. This ensured that larval growth was compared over a window when all fish were larvae. To determine mean larval growth for each year, we averaged increment widths of individuals from age 1 to 43 days, then computed the mean for each year. As size and age are tightly correlated, to test for differences in size-at-settlement across years after accounting for the effect of age we used ANCOVA with age as covariate. Because settlement of rockfish frequently included observations of zero individuals per SMURF, we used a generalized linear model with a negative binomial distribution to compare settlement across years. This analysis also included an offset variable to account for differences in the sampling interval between settlement observations^40^. We used Tukey-HSD to make pairwise comparisons of settlement across years.

To elucidate the contributions of basin-scale, regional, and local oceanographic conditions to interannual variability in growth and settlement, we utilized partial least squares regression (PLSR). PLSR is a multivariate analysis well suited to ecological data because it handles many colinear predictor variables, maximizes the variability explained in a response variable, is robust to small sample sizes^89^, and has been used to examine the influence of oceanographic conditions on fish growth^74^ and abundance^34^. We tested the influence of basin-scale oceanographic indices including PDO, ONI, NPGO, and NOI, regional values of BEUTI at 45^o^N, local oceanographic conditions including southern copepod index, sea surface temperature (SST), the meridional winds, as well as the spiciness index on black rockfish larval growth. Oceanographic conditions included in the PLSR were averages of the monthly values during the larval window for each year. We used these same conditions with the addition of the spiciness index to test the influence of oceanographic indicator variables on settlement magnitude.

We used simple linear regression to examine the relationship between the timing of metamorphosis from larval to pelagic juveniles and mean larval growth rate. A multiple regression model with linear and quadratic fits was used to determine whether settlement magnitude was correlated with larval growth.

All analyses were performed in R v4.1.2^75^ using packages dplyr and plsrdepot. Figures were generated using R package ggplot2.

## Supplementary Information

Supplementary Information.

## Data Availability

The data sets generated and/or analyzed during the current study are available in the Dryad data repository: https://doi.org/10.7291/D1109B.

## References

[1] Bindoff, N. L. *et al.* Changing ocean, marine ecosystems, and dependent communities. in *IPCC special report on the ocean and cryosphere in a changing climate* (eds. Pörtner, H.-O. et al.) (2019).

[2] Johnson GC, Lyman JM (2020). Warming trends increasingly dominate global ocean. Nat. Clim. Chang..

[3] Doney SC (2012). Climate change impacts on marine ecosystems. Ann. Rev. Mar. Sci..

[4] Pinsky ML, Mantua NJ (2014). Emerging adaptation approaches for climate- ready fisheries management. Oceanography.

[5] Bailey KM, Houde ED (1989). Predation on eggs and larvae of marine fishes and the recruitment problem. Adv. Mar. Biol..

[6] Houde ED (1989). Comparative growth, mortality, and energetics of marine fish larvae: temperature and implied latitudinal effects. Fish. Bull..

[7] Wang H, Shen S, Chen Y-S, Kiang Y-K, Heino M (2020). Life histories determine divergent population trends for fishes under climate warming. Nat. Commun..

[8] Llopiz JK (2014). Early life history and fisheries oceanography: New questions in a changing world. Oceanography.

[9] Lasker R (1975). Field criteria for survival of anchovy larvae: The relation between inshore chlorophyll maximum layers and successful first feeding. Fish. Bull..

[10] Cury P, Roy C (1989). Optimal environmental window and pelagic fish recruitment success in upwelling areas. Can. J. Fish. Aquat. Sci..

[11] Iles TD, Sinclair M (1982). Atlantic herring: Stock discreteness and abundance. Science.

[12] Houde ED (1987). Fish early life dynamics and recruitment variability. Am. Fish. Soc. Symp..

[13] Searcy SP, Sponaugle S (2001). Selective mortality during the larval – juvenile transition in two coral reef fishes. Ecology.

[14] Shima JS, Findlay AM (2002). Pelagic larval growth rate impacts benthic settlement and survival of a temperate reef fish. Mar. Ecol. Prog. Ser..

[15] Bakun A (1990). Global climate change and intensification of coastal ocean upwelling. Science.

[16] Snyder MA, Sloan L, Diffenbaugh N, Bell J (2003). Future climate change and upwelling in the California Current. Geophys. Res. Lett..

[17] Bakun A, Field DB, Redondo-Rodriguez A, Weeks SJ (2010). Greenhouse gas, upwelling-favorable winds, and the future of coastal ocean upwelling ecosystems. Glob. Chang. Biol..

[18] Bakun A, Nelson C (1991). The seasonal cycle of wind-stress curl in subtropical eastern boundary current regions. J. Phys. Oceanogr..

[19] Shanks AL, Eckert GL (2005). Population persistence of California Current fishes and benthic crustaceans: A marine drift paradox. Ecol. Monogr..

[20] Cushing DH (1990). Plankton production and year-class strength in fish populations: An update of the match/mismatch hypothesis. Adv. Mar. Biol..

[21] Carr MH (1991). Habitat selection and recruitment of an assemblage of temperate zone reef fishes. J. Exp. Mar. Bio. Ecol..

[22] Asch RG (2015). Climate change and decadal shifts in the phenology of larval fishes in the California Current ecosystem. Proc. Natl. Acad. Sci. U. S. A..

[23] Auth TD, Daly EA, Brodeur RD, Fisher JL (2018). Phenological and distributional shifts in ichthyoplankton associated with recent warming in the northeast Pacific Ocean. Glob. Chang. Biol..

[24] Sydeman WJ (2014). Climate change and wind intensification in coastal upwelling ecosystems. Science.

[25] Bond NA, Cronin MF, Freeland H, Mantua N (2015). Causes and impacts of the 2014 warm anomaly in the NE Pacific. Geophys. Res. Lett..

[26] Cavole A (2016). Biological impacts of the 2013–2015 warm water anomaly in the Northeast Pacific: Winner, losers, and the future. Oceanography.

[27] Lenarz, W. H. A history of California rockfish fisheries. In *Proceeding of the International Rockfish Symposium. Anchorage, Alaska, Univ. of Alaska* (1987).

[28] Brodeur RD, Buchanan JC, Emmett RL (2014). Pelagic and demersal fish predators on juvenile and adult forage fishes in the northern California Current: Spatial and temporal variations. CalCOFI Rep..

[29] Mills KL, Laidig T, Ralston S, Sydeman WJ (2007). Diets of top predators indicate pelagic juvenile rockfish (*Sebastes* spp.) abundance in the California Current System. Fish. Oceanogr..

[30] Santora JA, Schroeder ID, Field JC, Wells BK, Sydeman WJ (2014). Spatio-temporal dynamics of ocean conditions and forage taxa reveal regional structuring of seabird-prey relationships. Ecol. Appl..

[31] McClatchie S (2016). Food limitation of sea lion pups and the decline of forage off central and southern California. R. Soc. Open Sci..

[32] Love BMS, Yoklavich M, Thorsteinson L (2002). The Rockfishes of the Northeast Pacific.

[33] Ralston S, Howard DF (1995). On the development of year-class stength and cohort variability in two northern California rockfishes. Fish. Bull..

[34] Wells BK (2008). Untangling the relationships among climate, prey, and top predators in an ocean ecosystem. Mar. Ecol. Prog. Ser..

[35] Zabel RW, Levin PS, Tolimieri N, Mantua NJ (2011). Interactions between climate and population density in the episodic recruitment of bocaccio, *Sebastes paucispinis*, a Pacific rockfish. Fish. Oceanogr..

[36] Peterson WT (2014). Applied fisheries oceanography: Ecosystem indicators of ocean conditions inform fisheries management in the California Current. Oceanography.

[37] Wheeler SG, Anderson TW, Bell TW, Morgan SG, Hobbs JA (2016). Regional productivity predicts individual growth and recruitment of rockfishes in a northern California upwelling system. Limnol. Oceanogr..

[38] Ralston S, Sakuma KM, Field JC (2013). Interannual variation in pelagic juvenile rockfish (*Sebastes* spp.) abundance - going with the flow. Fish. Oceanogr..

[39] Schroeder ID (2019). Source water variability as a driver of rockfish recruitment in the california current ecosystem: Implications for climate change and fisheries management. Can. J. Fish. Aquat. Sci..

[40] Ottmann D, Grorud-Colvert K, Huntington B, Sponaugle S (2018). Interannual and regional variability in settlement of groundfishes to protected and fished nearshore waters of Oregon, USA. Mar. Ecol. Prog. Ser..

[41] Haggarty DR, Lotterhos KE, Shurin JB (2017). Young-of-the-year recruitment does not predict the abundance of older age classes in black rockfish in Barkley Sound, British Columbia. Canada. Mar. Ecol. Prog. Ser..

[42] Checkley DM, Barth JA (2009). Patterns and processes in the California Current System. Prog. Oceanogr..

[43] Jacox MG (2018). Forcing of multiyear extreme ocean temperatures that impacted California Current living marine resources in 2016. Bull. Am. Meteorol. Soc..

[44] Thompson AR (2019). Indicators of pelagic forage community shifts in the California Current Large Marine Ecosystem, 1998–2016. Ecol. Indic..

[45] Du X, Peterson WT (2018). Phytoplankton community structure in 2011–2013 compared to the extratropical warming event of 2014–2015. Geophys. Res. Lett..

[46] Peterson WT (2017). The pelagic ecosystem in the Northern California Current off Oregon during the 2014–2016 warm anomalies within the context of the past 20 years. J. Geophys. Res. Ocean..

[47] Brodeur RD, Auth TD, Phillips AJ (2019). Major shifts in pelagic micronekton and macrozooplankton community structure in an upwelling ecosystem related to an unprecedented marine heatwave. Front. Mar. Sci..

[48] Sutherland KR, Sorensen HL, Blondheim ON, Brodeur RD, Galloway AWE (2018). Range expansion of tropical pyrosomes in the northeast Pacific Ocean. Ecology.

[49] Brodeur RD, Hunsicker ME, Hann A, Miller TW (2019). Effects of warming ocean conditions on feeding ecology of small pelagic fishes in a coastal upwelling ecosystem: A shift to gelatinous food sources. Mar. Ecol. Prog. Ser..

[50] Bosley KL (2014). Feeding ecology of juvenile rockfishes off Oregon and Washington based on stomach content and stable isotope analyses. Mar. Biol..

[51] Reilly CA, Echeverria TW, Ralston S (1992). Interannual variation and overlap in the diets of pelagic juvenile rockfish (Genus: *Sebastes*) off central California. Fish. Bull..

[52] Sumida BY, Moser HG (1984). Food and feeding of bocaccio (*Sebastes paucispinis*) and comparison with Pacific hake (*Merluccius productus*) larvae in the California Current. Calif. Coop. Ocean. Fish. Investig. Reports.

[53] Auth TD, Brodeur RD, Soulen HL, Ciannelli L, Peterson WT (2011). The response of fish larvae to decadal changes in environmental forcing factors off the Oregon coast. Fish. Oceanogr..

[54] Frölicher TL, Laufkötter C (2018). Emerging risks from marine heat waves. Nat. Commun..

[55] Campana SE (1996). Year-class strength and growth rate in young Atlantic cod *Gadus morhua*. Mar. Ecol. Prog. Ser..

[56] Brander K (2000). Effects of environmental variability on growth and recruitment in cod (*Gadus morhua*) using a comparative approach. Oceanol. Acta.

[57] Sponaugle S, Grorud-Colvert K, Pinkard D (2006). Temperature-mediated variation in early life history traits and recruitment success of the coral reef fish *Thalassoma bifasciatum* in the Florida Keys. Mar. Ecol. Prog. Ser..

[58] Grorud-Colvert K, Sponaugle S (2011). Variability in water temperature affects trait-mediated survival of a newly settled coral reef fish. Oecologia.

[59] Boehlert GW, Yoklavich MM (1983). Effects of temperature, ration, and fish size on the growth of juvenile black rockfish, *Sebastes melanops*. Environ. Biol. Fishes.

[60] Chin B, Nakagawa M, Yamashita Y (2007). Effects of feeding and temperature on survival and growth of larval black rockfish *Sebastes schlegeli* in rearing conditions. Aquac. Sci..

[61] Woodbury D, Ralston S (1991). Interannual variation in growth rates and back-calculated birthdate distributions of pelagic juvenile rockfishes (*Sebastes* spp.) off the central California coast. Fish. Bull..

[62] Fennie H, Sponaugle S, Daly E, Brodeur R (2020). Prey tell: what quillback rockfish early life history traits reveal about their survival in encounters with juvenile coho salmon. Mar. Ecol. Prog. Ser..

[63] Laidig TE, Chess JR, Howard DF (2007). Relationship between abundance of juvenile rockfishes (*Sebastes* spp.) and environmental variables documented off northern California and potential mechanisms for the covariation. Fish. Bull..

[64] Robert D, Castonguay M, Fortier L (2007). Early growth and recruitment in Atlantic mackerel *Scomber scombrus*: discriminating the effects of fast growth and selection for fast growth. Mar. Ecol. Prog. Ser..

[65] Hare JA, Cowen RK (1997). Size, growth, development, and survival of the planktonic larvae of *Pomatomus saltatrix* (Pisces: Pomatomidae). Ecology.

[66] Takasuka A, Aoki I, Mitani I (2003). Evidence of growth-selective predation on larval Japanese anchovy *Engraulis japonicus* in Sagami Bay. Mar. Ecol. Prog. Ser..

[67] Anderson JT (1988). A review of size dependent survival during pre-recruit stages of fishes in relation to recruitment. J. Northwest Atl. Fish. Sci..

[68] Miller T, Crowder LB, Rice JA, Marschall EA (1988). Larval size and recruitment mechanisms in fishes: toward a conceptual framework. Can. J. Fish. Aquat. Sci..

[69] Chambers RC, Leggett WC (1987). Size and age at metamorphosis in marine fishes: analysis of laboratory-reared winter flounder (*Pseudopieuronectes americanus*) with a review of variation in other species. Can. J. Fish. Aquat. Sci..

[70] Kashef N, Sogard S, Fisher R, Largier J (2014). Ontogeny of critical swimming speeds for larval and pelagic juvenile rockfishes (*Sebastes* spp., family Scorpaenidae). Mar. Ecol. Prog. Ser..

[71] Paradis AR, Pepin P, Brown JA (1996). Vulnerability of fish eggs and larvae to predation: review of the influence of the relative size of prey and predator. Can. J. Fish. Aquat. Sci..

[72] Purcell JE (1989). Predation on fish larvae and eggs by the hydromedusa *Aequorea victoria* at a herring spawning ground in British Columbia. Can. J. Fish. Aquat. Sci..

[73] McLeod IM, Clark TD (2016). Limited capacity for faster digestion in larval coral reef fish at an elevated temperature. PLoS ONE.

[74] Takahashi M, Checkley DM, Litz MNC, Brodeur RD, Peterson WT (2012). Responses in growth rate of larval northern anchovy (*Engraulis mordax*) to anomalous upwelling in the northern California Current. Fish. Oceanogr..

[75] Team, R. C. R: A language and environment for statistical computing. (2013).

[76] Brady RX, Alexander MA, Lovenduski NS, Rykaczewski RR (2017). Emergent anthropogenic trends in California Current upwelling. Geophys. Res. Lett..

[77] Peterson WT, Keister JE (2003). Interannual variability in copepod community composition at a coastal station in the northern California Current: A multivariate approach. Deep Res. Part II Top. Stud. Oceanogr..

[78] Ammann AJ (2004). SMURFs: Standard monitoring units for the recruitment of temperate reef fishes. J. Exp. Mar. Bio. Ecol..

[79] Anderson TW, Carr MH (1998). BINCKE: A highly efficient net for collecting reef fishes. Environ. Biol. Fishes.

[80] Kilkenny C, Browne W, Cuthill IC, Emerson M, Altman DG (2010). Animal research: Reporting in vivo experiments: The ARRIVE guidelines. Br. J. Pharmacol..

[81] Laidig, T. E. & Adams, P. B. *Methods used to identify pelagic juvenile rockfish (Genus* Sebastes*) occuring along the coast of central California*. *NOAA Technical Memorandum NMFS* (1991).

[82] Di Lorenzo E, Mantua N (2016). Multi-year persistence of the 2014/15 North Pacific marine heatwave. Nat. Clim. Chang..

[83] Yoklavich MM, Boehlert GW (1987). Daily growth increments in otoliths of juvenile black rockfish, *Sebastes melanops*: An evaluation of autoradiography as a new method of validation. Fish. Bull..

[84] Miller JA, Shanks AL (2004). Evidence for limited larval dispersal in black rockfish (*Sebastes melanops*): Implications for population structure and marine-reserve design. Can. J. Fish. Aquat. Sci..

[85] Sponaugle S, Green B, Mapstone B, Carlos G, Begg G (2009). Daily otolith increments in the early stages of tropical fish. Tropical Fish Otoliths: Information for Assessment, Management and Ecology.

[86] Laidig T, Ralston S, Bence JR (1991). Dynamics of growth in the early life history of shortbelly rockfish *Sebastes jordani*. Fish. Bull..

[87] Thorrold SR, Hare JA, Sale PF (2002). Otolith applications in reef fish ecology. Coral Reef Fishes: Dynamics and Diversity in a Complex Ecosystem.

[88] Field JC, MacCall AD, Ralston S, Love MS, Miller EF (2010). Bocaccionomics: The effectiveness of pre-recruit indices for assessment and management of bocaccio. Calif. Coop. Ocean. Fish. Investig. Reports.

[89] Carrascal LM, Galván I, Gordo O (2009). Partial least squares regression as an alternative to current regression methods used in ecology. Oikos.

