# Supplementary material for: Larval rockfish growth and survival in response to anomalous ocean conditions
Source: Sci Rep. 2023 Mar 11;13:4089. doi: 10.1038/s41598-023-30726-5 (PMC10008550; doi:10.1038/s41598-023-30726-5)

**Supplementary Materials**

**Table S1.** Summary of previous studies examining the relationships between pelagic juvenile rockfish (*Sebastes* spp.) abundance and oceanographic conditions. Each of these studies used rockfishes collected off central California.

| Study | Species | Years | Environmental variables | Recruitment pattern | Life stage |
| --- | --- | --- | --- | --- | --- |
| Ralston & Howard 1995 | S. flavidus,  S. mystinus | 1983-1992 | Winter water temperature | Dome shaped relationship with water temperature and recruitment | Pelagic juvenile  Settled juvenile |
| Laidig et al. 2007 | S. flavidus,  S. melanops,  S. mystinus | 1983-2003 | Winter sea level anomaly and nearshore temperature | Negatively correlated with winter sea level anomaly and nearshore temperature | Settled juvenile |
| Wells et al. 2008 | S. jordani | 1975-2005 | Zooplankton abundance and wind stress curl | Positively correlated with zooplankton and negatively correlated with curl | Pelagic juvenile  Settled juvenile |
| Zabel 2011 | S. paucispinis | 1968-2006 | Northern Oscillation Index | Positively correlated with NOI | Pelagic juvenile  Settled juvenile |
| Ralston et al. 2013 | S. auriculatus,  S. entomelas,  S. flavidus,  S. goodei,  S. hopkinsi,  S. jordani,  S. mystinus,  S. paucispinis,  S. pinniger,  S. saxicola | 1983-2010 | Sea level anomalies | Increased recruitment when sea level anomalies indicate equatorward flow during the larval stage | Pelagic juvenile  Settled juvenile |
| Schroeder et al. 2014 | S. auriculatus,  S. entomela,  S. flavidus,  S. goodei,  S. hopkinsi,  S. jordani,  S. mystinus,  S. paucispinis,  S. pinniger,  S. saxicola | 1990-2010 | Isopycnal depth | Negatively correlated with isopycnal depth | Pelagic juvenile  Settled juvenile |
| Wheeler et al. 2016 | S. carnatus,  S. caurinus | 2010-2011 | Regional Productivity | Positively correlated with productivity | Settled juvenile |
| Schroeder et al. 2019 | S. auriculatus, S. entomela, S. flavidus,  S. goodei,  S. hopkinsi,  S. jordani,  S. mystinus,  S. paucispinis, S. pinniger,  S. saxicola | 1983-2016 | "Spiciness index" | Positively correlated with Pacific Subarctic water signature in upper waters | Pelagic juvenile |

Figure S1. Comparison of daily larval growth trajectories between the one juvenile black rockfish collected in central Oregon (black) and the five juvenile black rockfish collected from southern Oregon (grey) that were used to increase the sample size of black rockfish growth rates in 2015.


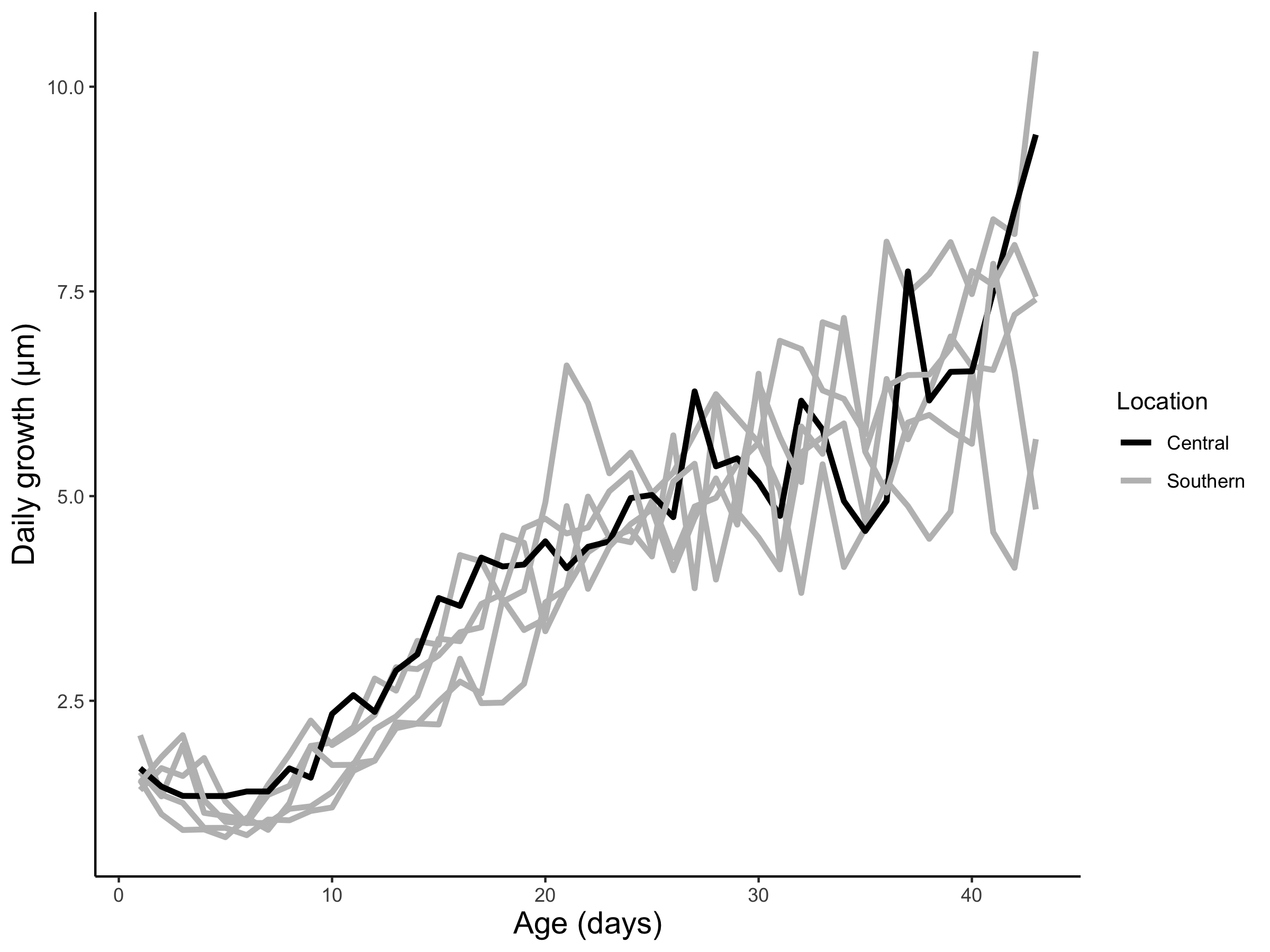


Figure S2. Comparison of interannual variability in daily larval growth trajectories between juvenile black rockfish collected in 2013 (blue) and 2015 (red).


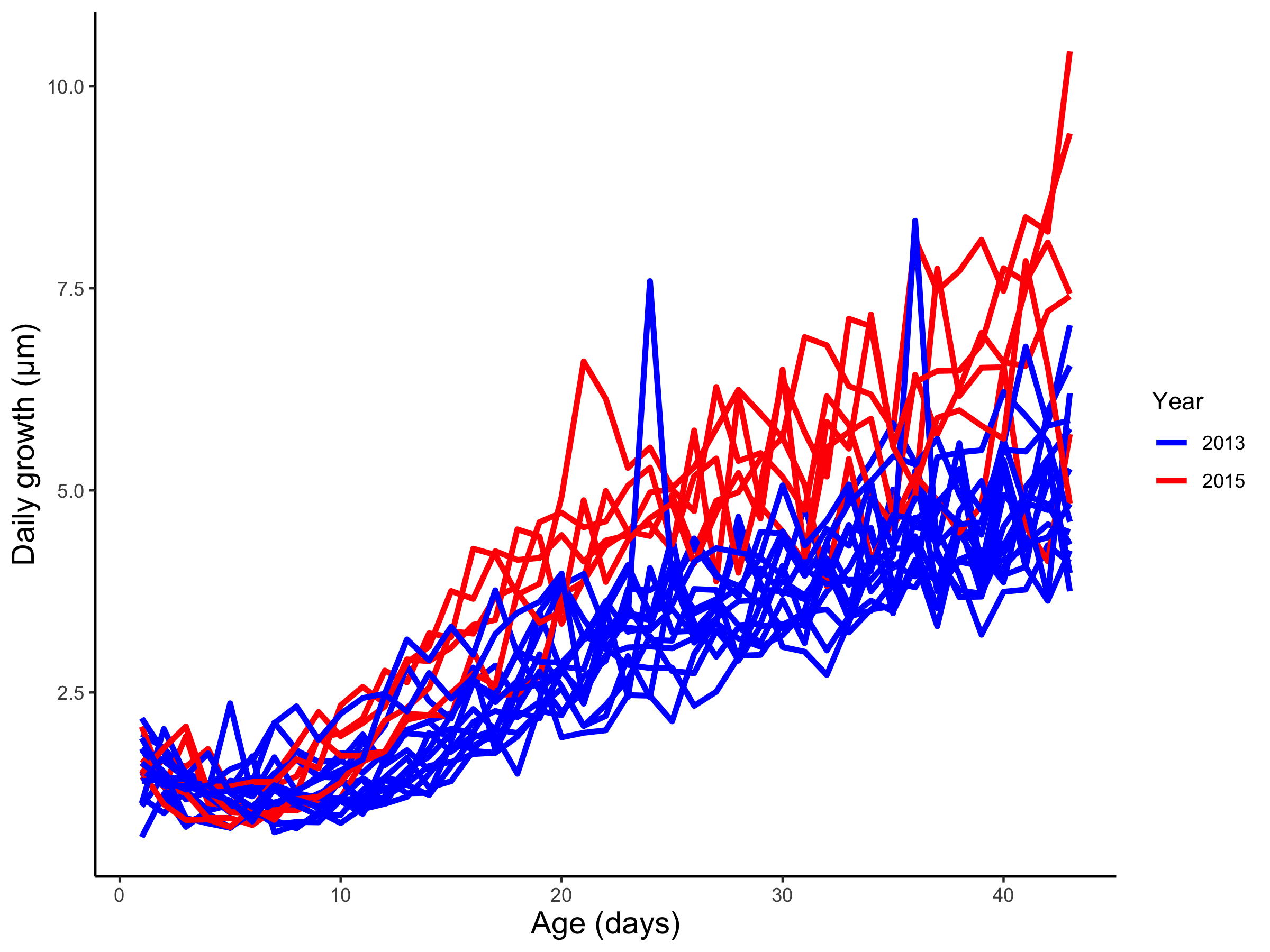


Figure S3. Multiple regression of mean settlement rate of black rockfish (*Sebastes melanops*) to coastal Oregon as measured in replicate Standardized Monitoring Units for the Recruitment of Fishes (SMURFs) over 6 years (excluding 2015) as a function of mean larval growth and mean larval growth^2^. Gray area is 95% confidence interval.


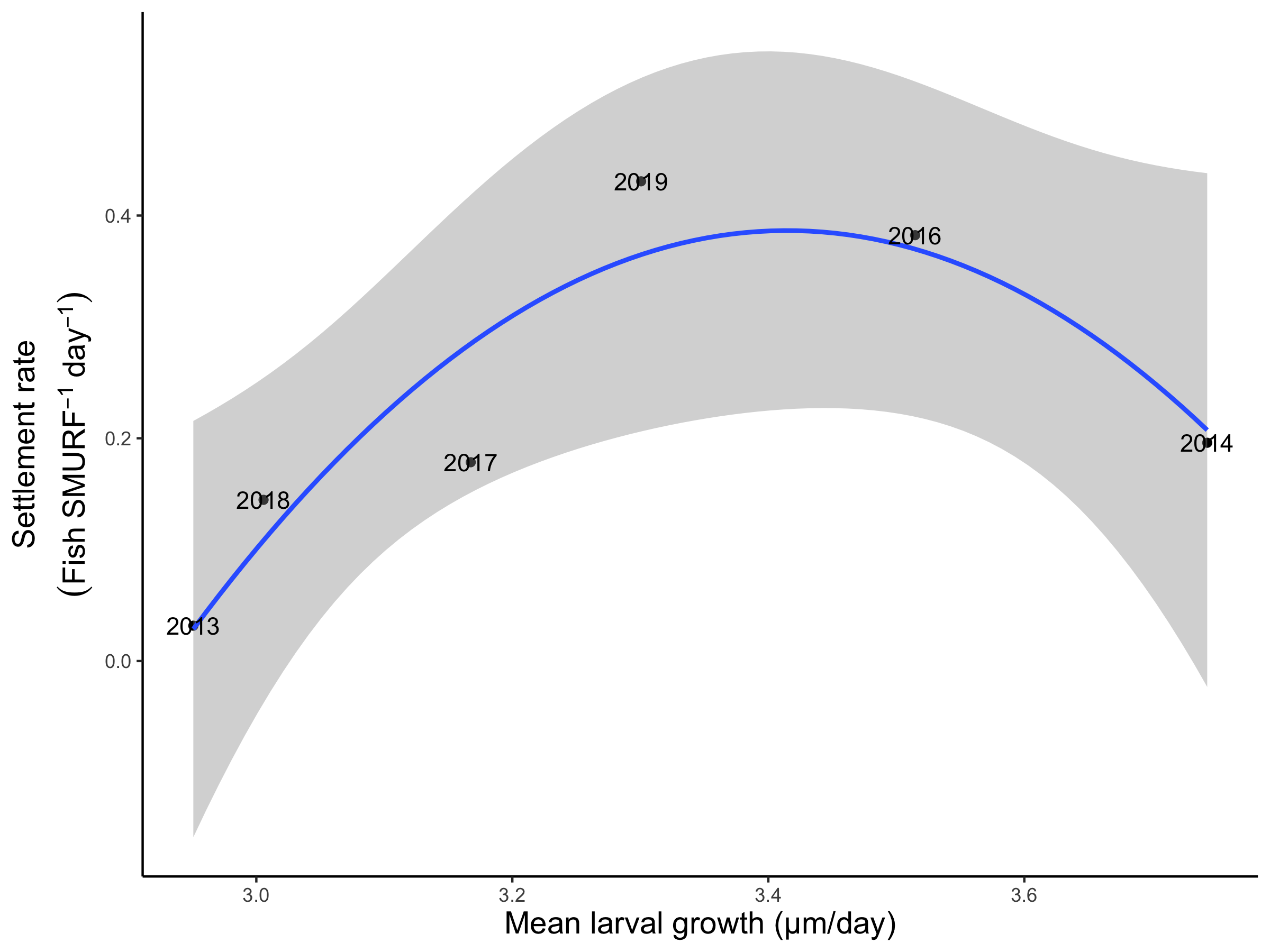

Supplement: Supplementary file 1 — Supplementary Information. [file 41598_2023_30726_MOESM1_ESM.docx]
